# Artificial intelligence-based refractive error prediction and EVO-implantable collamer lens power calculation for myopia correction

**DOI:** 10.1186/s40662-023-00338-1

**Published:** 2023-05-01

**Authors:** Yinjie Jiang, Yang Shen, Xun Chen, Lingling Niu, Boliang Li, Mingrui Cheng, Yadi Lei, Yilin Xu, Chongyang Wang, Xingtao Zhou, Xiaoying Wang

**Affiliations:** 1 Eye Ear Nose and Throat Hospital, Fudan University, No. 19 BaoQing Road, XuHui District, Shanghai, 200031 China; 2 National Health Commission Key Lab of Myopia, Fudan University, Shanghai, China; 3 Shanghai Research Center of Ophthalmology and Optometry, Shanghai, China; 4 Research and Development Department, Shanghai MediWorks Precision Instruments Company Limited, Shanghai, China

**Keywords:** Artificial intelligence, Machine learning, Refractive error, Myopia, Implantable collamer lens, Toric implantable collamer lens, Lens power calculation

## Abstract

**Background:**

Implantable collamer lens (ICL) has been widely accepted for its excellent visual outcomes for myopia correction. It is a new challenge in phakic IOL power calculation, especially for those with low and moderate myopia. This study aimed to establish a novel stacking machine learning (ML) model for predicting postoperative refraction errors and calculating EVO-ICL lens power.

**Methods:**

We enrolled 2767 eyes of 1678 patients (age: 27.5 ± 6.33 years, 18–54 years) who underwent non-toric (NT)-ICL or toric-ICL (TICL) implantation during 2014 to 2021. The postoperative spherical equivalent (SE) and sphere were predicted using stacking ML models [support vector regression (SVR), LASSO, random forest, and XGBoost] and training based on ocular dimensional parameters from NT-ICL and TICL cases, respectively. The accuracy of the stacking ML models was compared with that of the modified vergence formula (MVF) based on the mean absolute error (MAE), median absolute error (MedAE), and percentages of eyes within ± 0.25, ± 0.50, and ± 0.75 diopters (D) and Bland-Altman analyses. In addition, the recommended spheric lens power was calculated with 0.25 D intervals and targeting emmetropia.

**Results:**

After NT-ICL implantation, the random forest model demonstrated the lowest MAE (0.339 D) for predicting SE. Contrarily, the SVR model showed the lowest MAE (0.386 D) for predicting the sphere. After TICL implantation, the XGBoost model showed the lowest MAE for predicting both SE (0.325 D) and sphere (0.308 D). Compared with MVF, ML models had numerically lower values of standard deviation, MAE, and MedAE and comparable percentages of eyes within ± 0.25 D, ± 0.50 D, and ± 0.75 D prediction errors. The difference between MVF and ML models was larger in eyes with low-to-moderate myopia (preoperative SE >  − 6.00 D). Our final optimal stacking ML models showed strong agreement between the predictive values of MVF by Bland-Altman plots.

**Conclusion:**

With various ocular dimensional parameters, ML models demonstrate comparable accuracy than existing MVF models and potential advantages in low-to-moderate myopia, and thus provide a novel nomogram for postoperative refractive error prediction and lens power calculation.

**Supplementary Information:**

The online version contains supplementary material available at 10.1186/s40662-023-00338-1.

## Key points

- We constructed novel stacking machine learning models based on preoperative dimensional ocular parameters of 2767 eyes of 1678 patients who underwent non-toric or toric ICL implantation.
- The final optimal models predicted postoperative spherical equivalent and sphere with numerically higher accuracy and showed strong agreement with the existing modified vergence formula.
- The stacking machine learning models demonstrated potential advantages with various ocular dimensional parameters in predicting postoperative refractive error and provided a novel nomogram to calculate phakic intraocular lens power.

**Supplementary Information:**

The online version contains supplementary material available at 10.1186/s40662-023-00338-1.

## Background

Refractive errors are major contributors to reversible visual impairment globally [1]. Myopia has become a serious global public health concern because of its rising prevalence [2]. Although corneal refractive surgeries have dominated the field of refractive surgery in recent decades, the corneal thickness and keratometry restrict the amount of refractive error correction. Phakic intraocular refractive surgeries provide surgical options for individuals unsuitable for corneal refractive surgery, such as extremely severe ametropia [3]. Nowadays, the implantable collamer lens (ICL, type V4C; STAAR Surgical, Monrovia, CA, USA), including the non-toric ICL (NT-ICL) and toric ICL (TICL), is the most widely used posterior chamber phakic intraocular lenses (IOL). The implantation of ICL can correct a wide range of myopia (up to − 18.00 D) [4] and severe astigmatism (up to 6.00 D) [5] or even stable keratoconus [6]. In addition, the central hole design minimizes the risk of complications associated with the old generation of phakic IOLs [7, 8]. The potential advantage has been widely accepted, including fast visual recovery, stable postoperative refractive outcome, and excellent visual quality [4]. However, refractive surprises still occur in a few cases. Packer’s meta-analysis demonstrated that the postoperative residual refraction error over 0.50 D and 1.00 D to the target were 9.1% (1.5%–28%) and 1.3% (0%–8.3%), respectively [8]. Montés-Micó et al. found that the corresponding rates were even higher [7]. Given the sheer volume of ICL surgery worldwide (over one million lenses have been sold during the past three years globally[9]), most patients of ICL surgery are young and have high expectations of the visual outcomes. Therefore, the 1% of patients with refractive surprise would likely be clinically relevant at a population level.

An accurate calculation of the ICL lens power is critical to ensure satisfactory refractive and visual outcomes. The Van Der Heijdei and Holladay formula has historically been used to calculate phakic lens power [10]. Matrix schemes were also used in toric phakic IOL lens power [11, 12]. According to the vergence formula, dimensional ocular parameters may affect the postoperative refractive error, including anterior chamber depth, corneal curvature [13], and postoperative lens position [14–16]. Vault, defined as the distance between the posterior surface of the ICL lens and the anterior surface of the crystalline lens, is used to reflect the ICL lens position. Previous studies have found that higher vault tends to have hyperopia and lower vault tends to have myopia [14–16]. Others have also reported that postoperative refractive outcome after ICL implantation depends on preoperative refraction [17–19]. Therefore, the more accurate lens power step may help improve the accuracy of refractive correction. Currently, the EVO-ICL lens power step of ICL is 0.25 D in less than + 3.00 D and 0.50 D in over + 3.00 D, as those with low myopia may have less tolerance to refractive prediction error (PE). The sphere lens power step of EVO TICL is 0.50 D range from + 0.50 D to + 18.00 D [20]. Therefore, it is a new challenge in phakic IOL power calculation, especially for those with low and moderate myopia.

Artificial intelligence (AI) has recently performed excellently, optimizing new-generation aphakic IOL lens calculation models [21–23]. In contrast, machine learning (ML) has various advanced models that can explore the relationship between many variables [24]. This study aimed to establish and verify novel stacking ML models based on a large dataset for predicting postoperative refraction errors of ICL implantation to improve the predictability of postoperative refractive outcomes after ICL implantation. Furthermore, we developed an ICL lens sphere calculator based on optimal ML models for clinical applications.

## Methods

### Ethics statements

The Ethics Committee of the Eye and ENT Hospital of Fudan University (No. 2021018) approved the study, and all study procedures adhered to the tenets of the Declaration of Helsinki. Each participant provided informed consent after receiving a detailed explanation of the procedure before treatment. In this study, patient-identifiable data were hidden.

### Study design and population

This retrospective study enrolled patients who underwent uneventful NT-ICL (type V4C; STAAR Surgical) or TICL (type V4C; STAAR Surgical) implantation during 2014 and 2021 at the Eye and ENT Hospital of Fudan University, Shanghai, China. All procedures were performed by two experienced surgeons (XYW and XTZ) with experience in over 1,000 ICL implantation. The exclusion criteria were as follows: (1) A history of other ocular diseases or surgery; (2) Postoperative decimal BCVA < 0.5 (10/20); (3) Preoperative astigmatism > 0.50 D but NT-ICL implantation [those who may correct astigmatism through surgical induced astigmatism (SIA)]; (4) Perioperative or postoperative complications (including realignment or exchange); or (5) Incomplete follow-up medical records.

### Measurements

All patients underwent routine preoperative assessments, including uncorrected distance visual acuity (UDVA), manifest refraction, and best-corrected distance visual acuity (BDVA). In addition, axial length (AL) was measured using an optical biometer (IOL-master 1322-734; Carl Zeiss Meditec AG, Jena, Germany); intraocular pressure (IOP) was measured using a non-contact tonometer (Canon Full Auto Tonometer TX-F; Canon, Tokyo, Japan); and scotopic pupil diameter (PD) was evaluated using an auto-refractor (ARK-1, NIDEK, Aichi, Japan) under scotopic conditions. In addition, the main anterior segment parameters, including the steepest keratometry (K1), K1 axis, flattest keratometry (K2), K2 axis, anterior chamber depth (ACD), anterior chamber angle (ACA), PD, corneal thickness (CT), horizontal corneal diameter [white-to-white (WTW)], and postoperative vault values, were obtained using an anterior segment analyzer (Pentacam HR; OCULUS, Wetzlar, Germany). An experienced technician (LLN) prescribed all ICL sizes and lens powers. The criteria to determine TICL were: (1) Difference between the best-corrected visual acuity (BCVA) with cylindrical lenses and spherical lenses alone is more than two lines; (2) Astigmatism >  − 1.00 D or astigmatism to spherical ratio > 1/3; (3) Patients who have never been fitted with corneal contact lenses and who wear frame glasses with cylindrical lenses for long periods of time; (4) Patients who are not suitable for surgically induced astigmatism (SIA) [the difference of K2 axis measured by corneal topography (Pentacam) and axis by manifest refraction over 15°] but have high requirements for visual outcomes; (5) Patients with factors related to worse predictability of vault (ACD < 2.8 mm) were not recommended TICL. All the lens power designs were on trial in frame glasses preoperatively and accepted by the patients. Data were obtained preoperatively, and at the last follow-up during postoperative one week and one month.

### Modelling

**Fig. 1:**
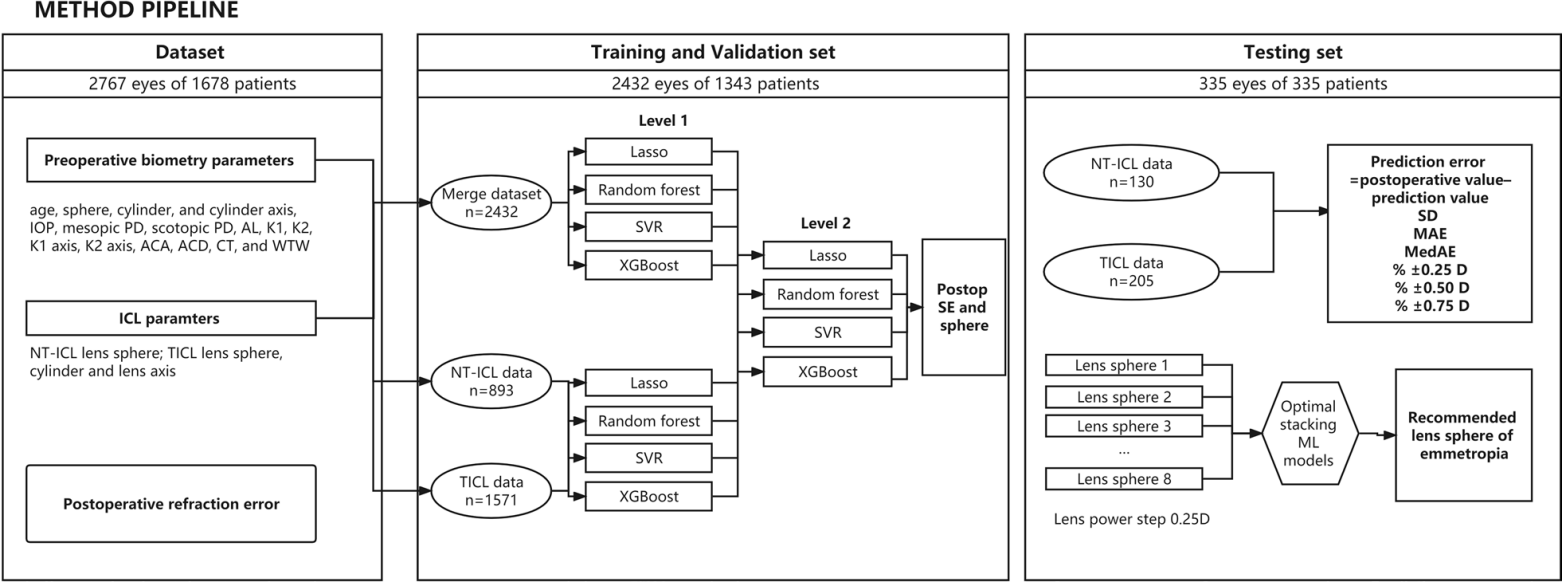
Flowchart of our study. NT-ICL, non-toric implantable collamer lens; TICL, toric implantable collamer lens; IOP, intraocular pressure; PD, pupil diameter; AL, axial length; K1, steepest keratometry; K2 flattest keratometry; ACA, anterior chamber angle; ACD, anterior chamber depth; CT, corneal thickness; WTW, white-to-white; SVR, support vector regression; SD, standard deviation; MAE, mean absolute error; MedAE, median absolute error; ML, machine learning

Figure 1 shows the steps involved in constructing the models. In total, data from 4150 eyes were collected. After exclusion, 3085 eyes of 1678 patients were included (age: 27.5 ± 6.33 years, 18–54 years). Before building a ML algorithm, the data were randomly divided into the training/validation set and testing set in a 4:1 ratio. One eye was randomly removed from the dataset if both eyes were in the testing set to ensure the independence of all samples in the testing set and prevent ‘both eye bias’. Finally, 2432 eyes of 1343 patients were arranged in the training/validation set, and 335 eyes were arranged in the testing set.

The postoperative spherical equivalent (SE) and sphere were outputs (predicted values). The input parameters included age, preoperative refraction (sphere, cylinder, and cylinder axis), ophthalmologic biometry (IOP, mesopic PD, scotopic PD, AL, K1, K2, K1 axis, K2 axis, ACA, ACD, CT, and WTW), and ICL parameters (NT-ICL: lens sphere; TICL: lens sphere, cylinder and lens axis).

Four ML models were trained to predict postoperative SE and sphere, including support vector regression (SVR), LASSO, random forest (RF), and XGBoost. We used a stacking method to take advantage of the different classes of ML models. The stacking process consists of three steps and two levels (two steps on level 1 and one step of stacking on level 2). First, ML models were trained based on merging NT-ICL and TICL data to capture similar features of NT-ICL and TICL. The cylinder and axis of NT-ICL were set to zero. Then, ML models were trained based on the NT-ICL or TICL datasets separately to learn more about the different features of NT-ICL and TICL. Finally, the trained models of the first level were used to perform cross-prediction on the training/validation set and average prediction on the testing set, and the prediction results were used as newly added input parameters.

In the training/validation set, five-fold cross-validation with a grid search was used to optimize the hyperparameters for training models. The split of the training and validation data followed the uniocular principle. In addition, impurity-based feature importance was used to investigate the importance of the outputs and inputs in the training/validation set.

### Outcome measurement and evaluation of models using the testing sets

The outputs from level 2 stacking models were the final prediction results. We compared the prediction performance between level 2 stacking models and the result of the ICL Power Calculation software provided by the manufacturer (version 3.0, http://en.informer.com/icl-power-calculationsoftware/), which is based on the modified vergence formula (MVF). Each model’s PE was calculated for each eye (PE = postoperative manifest refraction value − prediction value by stacking ML models or MVF). A negative PE represents a myopic error while a positive PE represents a hyperopic error. The evaluation metrics included the mean prediction error (MPE) and its standard deviation (SD), mean absolute error (MAE), median absolute error (MedAE), and percentage of eyes with a PE within ± 0.25 D, ± 0.50 D, and ± 0.75 D. The stacking ML models with the smallest MAE were selected as the optimal models [if the MAEs were similar, we compared the SD, MedAE, and absolute error (AE) quartile].

### Application of lens power calculator

Finally, optimal ML models were used to determine the most appropriate NT-ICL and TICL sphere values by enumerating the lens sphere at an interval of 0.25 D and targeting emmetropia. Next, the NT-ICL and TICL PE (lens PE) were calculated for each eye (lens PE = implanted ICL sphere − recommended lens sphere). We calculated the percentage of eyes with lens PE by a specified amount (0.25 D, 0.50 D, and 0.75 D) to reflect the frequency at which the surgeon would have to make a potentially different clinical choice. A three-dimensional (3D) surface fitted represented the regions where the recommended lens sphere by stacking ML models differed from the implanted lens sphere. Finally, the correlation between the lens PE, preoperative sphere, and age was demonstrated.

### Statistical analysis

All statistical analyses were performed using SPSS (version 25.0; IBM Corp., Armonk, NY, USA). Unpaired t-tests were used to compare the average values of continuous variables between the training and test data. The normality of the PE was assessed using the Kolmogorov-Smirnov test. The statistical analysis of predictive outcomes follows the protocols recommended by Wang et al. [25] In the testing set of NT-ICL and TICL cases, we adjusted the MPE of different models to zero to eliminate the systematic error caused by the clinical environment. The Friedman test was used to compare MAEs. The Cochran Q test was used to compare the percentage of eyes with a PE within ± 0.25 D, ± 0.50 D, and ± 0.75 D. According to Holladay’s recommendation, the distribution of PE was demonstrated as a density-histogram graph to analyze further the distribution of SE and sphere PE [26]. The agreement of the lens sphere given by the best stacking ML models and the MVF were evaluated using Bland-Altman analyses. The subgroup analysis was performed in a different range of myopia (low-to-moderate myopia: preoperative SE ≥  − 6.00 D, high myopia: − 10.00 D ≤ preoperative SE < − 6.00 D, super high myopia: preoperative myopia: preoperative SE ≤ − 10.00 D). The correlation between the lens sphere PEs of ML models, age and preoperative SE was evaluated using Pearson correlation analyses. The cut-off *P *value was less than 0.05.

## Results

### Study population and characteristics

For NT-ICL cases, 893 eyes of 575 patients were included in the training/validation set, and 130 eyes of 130 patients were included in the testing set. For TICL cases, 1571 eyes of 911 patients were included in the training/validation set, and 205 eyes of 205 patients were included in the testing set. Patient characteristics were similar between the training and testing datasets of NT-ICL and TICL (Table 1).

**Table 1:** Baseline demographics

	NT-ICL cases				TICL cases			
Characteristics	Training/validation set (n = 893)		Testing set (n = 130)		Training/validation set (n = 1571)		Testing set (n = 205)	
	n/mean	%/SD	n/mean	%/SD	n/mean	%/SD	n/mean	%/SD
Left eye, n (%)	414	46.36%	60.00	46.15%	806	51.30%	90	43.90%
Female, n (%)	730	81.75%	106.00	81.54%	1208	76.89%	157	76.59%
Age (years)	28.78	6.53	28.78	6.37	26.78	6.10	26.85	6.20
Preop SE (D)	− 9.52	3.75	− 9.08	3.82	− 10.07	3.12	− 9.91	2.75
Preop S (D)	− 9.46	3.72	− 8.99	3.71	− 9.10	3.02	− 8.92	2.71
Preop C (D)	− 0.12	0.32	− 0.18	0.53	− 1.94	0.95	− 1.98	0.87
Preop A (degree)	20.55	47.92	30.35	59.61	113.60	76.69	98.17	81.08
BCVA (logMAR)	0.02	0.11	0.02	0.09	0.02	0.08	0.01	0.08
Mesopic PD (mm)	6.75	0.74	6.69	0.71	6.82	0.74	6.90	0.72
Photopic PD (mm)	3.19	0.59	3.25	0.62	3.20	0.60	3.22	0.62
IOP (mmHg)	15.37	2.66	15.02	2.47	15.60	2.69	15.60	2.71
Axial length (mm)	27.40	1.77	27.18	1.76	27.38	1.45	27.37	1.39
K1 (D)	42.94	1.47	42.77	1.30	42.81	1.39	42.82	1.48
K1 axis (degree)	88.69	77.02	107.72	75.23	93.36	81.46	80.75	82.56
K2 (D)	43.83	1.56	43.65	1.44	44.73	1.55	44.83	1.73
K2 axis (degree)	90.60	21.68	91.10	22.81	89.87	15.64	91.73	10.69
Kmean (D)	43.39	1.49	43.21	1.35	43.77	1.42	43.82	1.54
ACD (mm)	3.18	0.24	3.15	0.25	3.23	0.24	3.22	0.24
CT (mm)	0.52	0.03	0.53	0.03	0.53	0.03	0.53	0.03
WTW (mm)	11.57	0.39	11.55	0.32	11.63	0.36	11.65	0.35
ACV (mm^3^)	199.49	32.30	198.25	32.40	203.24	30.42	202.86	31.27
ACA (degree)	38.53	5.13	37.74	5.47	38.95	5.21	38.65	5.17
ICL S (D)	− 10.15	3.20	− 9.67	3.25	− 11.79	3.00	− 11.64	2.62
ICL C (D)	NA	NA	NA	NA	1.88	0.93	1.92	0.85
ICL A (degree)	NA	NA	NA	NA	89.75	19.02	89.99	14.77
ICL length (mm), n (%)								
12.1 mm	108	12.09%	15.00	11.54%	142	9.04%	23	11.22%
12.6 mm	443	49.61%	72.00	55.38%	769	48.95%	89	43.41%
13.2 mm	288	32.25%	41.00	31.54%	593	37.75%	81	39.51%
13.7 mm	54	6.05%	2.00	1.54%	67	4.26%	12	5.85%
Postop SE (D)	− 0.34	0.76			− 0.26	0.57	− 0.21	0.49
Postop S (D)	− 0.03	0.68	0.05	0.70	0.01	0.55	0.06	0.47
Postop C (D)	− 0.62	0.54	− 0.62	0.52	− 0.54	0.40	− 0.54	0.44
Postop A (degree)	72.72	71.65	68.47	72.40	59.56	67.90	53.00	65.01
Postop BCVA (logMAR)	− 0.04	0.08	− 0.04	0.07	− 0.04	0.07	− 0.04	0.07

*preop* = preoperative; *SE* = spherical equivalent; *S* = sphere; *C* = cylinder; *A* = axis; *D* = diopters; *BCVA* = best-corrected visual acuity; *mesopic* *PD* = mesopic pupil diameter obtained using auto-refraction; *photopic* *PD* = photopic pupil diameter obtained using Pentacam HR; *IOP* = intraocular pressure; *K1* = steepest keratometry; *K2* = flattest keratometry; *ACD* = anterior chamber depth; *CT* = corneal thickness; *WTW* = white to white; *ACV* = anterior chamber volume; *ACA* = anterior chamber angle; *NA* = not applicable; *postop* = postoperative

### Importance of features

**Fig. 2:**
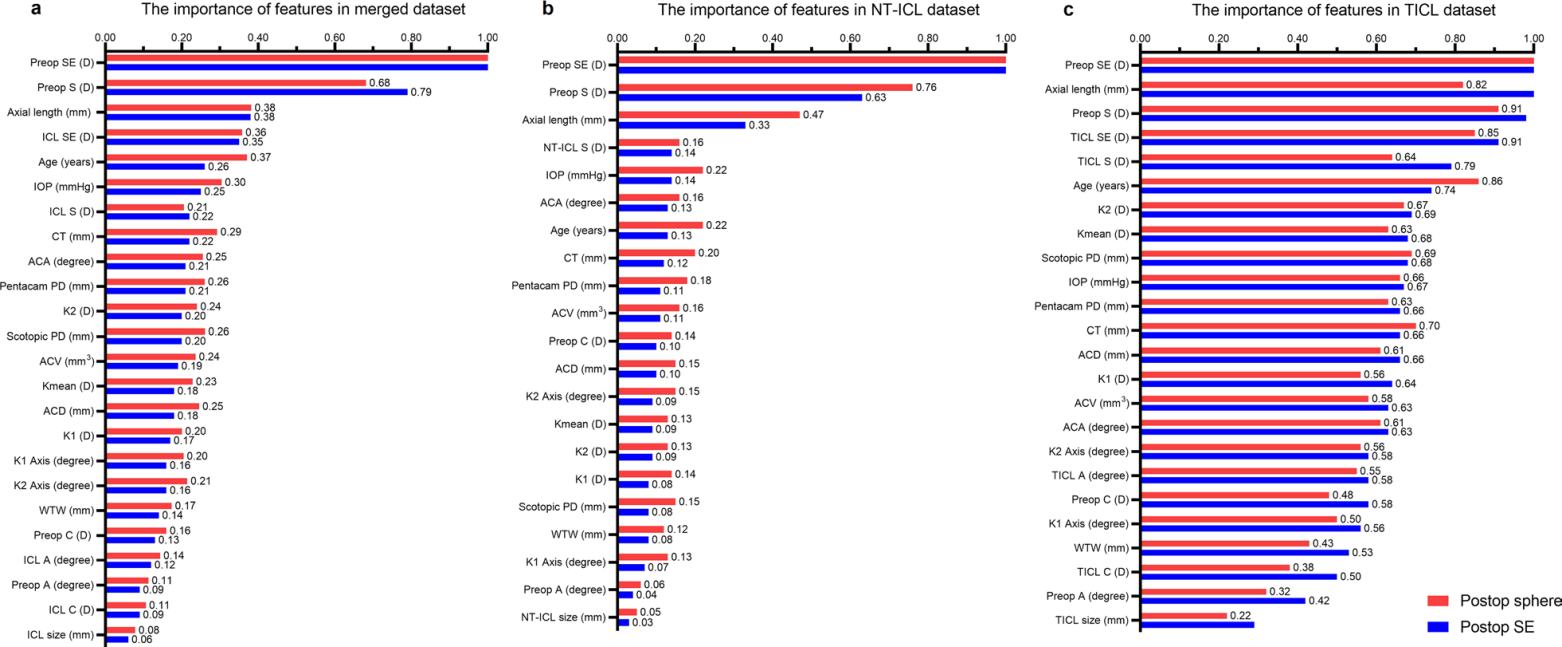
Importance of features for predicting postoperative spherical equivalent (SE) and sphere. **a** Importance of features in a merged dataset; **b** Importance of features in the NT-ICL dataset; **c** Importance of features in the TICL dataset. The cylinder and axis of the NT-ICL were set to 0.00. The highest importance is aligned to 1.00 and scales the left values accordingly. ICL, implantable collamer lens; NT-ICL, non-toric ICL; TICL, toric ICL; preop, preoperative; S, sphere; C, cylinder; A, axis; D, diopters; scotopic PD, pupil diameter obtained using an auto-refractor; Pentacam PD, pupil diameter obtained using Pentacam HR; IOP, intraocular pressure; K1, steepest keratometry; K2, flattest keratometry; Kmean, mean keratometry; ACD, anterior chamber depth; CT, corneal thickness; WTW, white to white; ACV, anterior chamber volume; ACA, anterior chamber angle

Before stacking, the importance of parameters in the ML models trained based on merged datasets and separately is shown in Fig. 2. When ML models were trained based on merged datasets, the importance of preoperative SE was the highest for predicting postoperative SE and sphere (both importance = 1.00) followed by the preoperative sphere (importance = 0.79 for predicting SE and 0.68 for predicting sphere) and AL (importance = 0.38 for predicting SE; 0.38 for predicting sphere; Fig. 2a). When training separately, the three metrics were the top three most important for the postoperative refraction prediction of either NT-ICL or TICL (Fig. 2b, c). Except for ICL size (which was not as important as other metrics), the importance of all the other parameters in the TICL dataset was higher than that in the NT-ICL dataset for predicting postoperative SE and sphere.

### Prediction performance of the stacking ML models

**Fig. 3:**
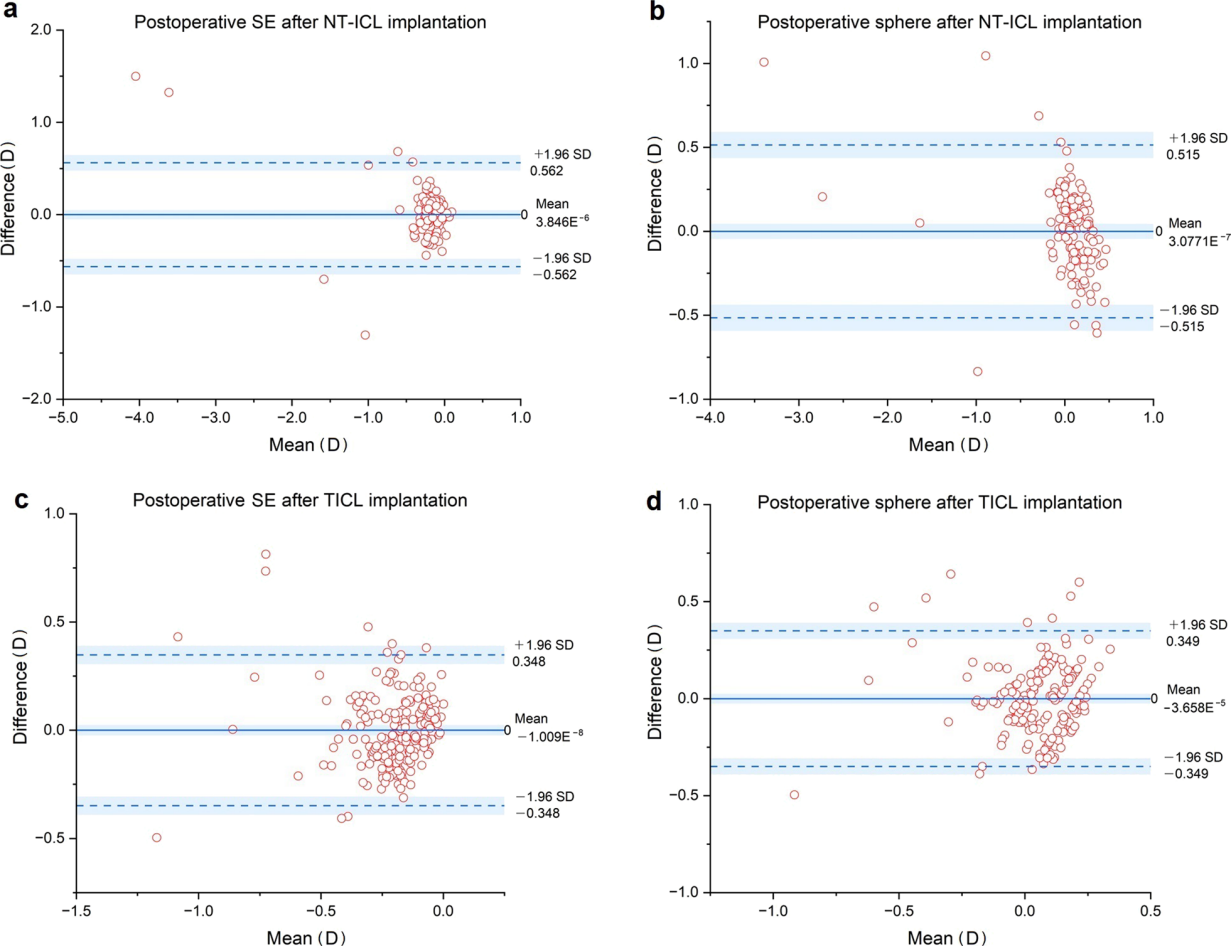
Bland-Altman plot of agreement between the predicted values of machine learning (ML) models and the modified vergence formula (MVF). **a** Bland-Altman plot of postoperative SE after NT-ICL implantation; **b** Bland-Altman plot of postoperative sphere after NT-ICL implantation; **c** Bland-Altman plot of postoperative sphere after TICL implantation; **d** Bland-Altman plot of postoperative sphere after TICL implantation. The mean difference (red) and 95% limits of agreement (mean difference − 1.96 SD, mean difference + 1.96 SD, black) are indicated by dotted lines. NT-ICL, non-toric implantable collamer lens; TICL, toric implantable collamer lens

All parameters were used in the stacking ML models. Table 2 demonstrates the prediction performance in the testing set of NT-ICL and TICL cases, respectively. The optimal stacking ML models based on MAE rankings were the RF for SE prediction, SVR for postoperative sphere prediction (MAE = 0.386 D) after NT-ICL implantation, and XGBoost for postoperative SE prediction (MAE = 0.325 D) and sphere prediction (MAE = 0.308 D) after TICL implantation (Table 1). Compared to the MVF, the best stacking ML models had numerically lower SD, MAE, MedAE, and AE quartile values in all predictions*.* However, the distribution of absolute PEs did not differ significantly between all the models and the MVF (Friedman test in Additional file 1). The *P* value of the PE distribution of postoperative SE after TICL implantation was 0.014. The distribution of the PE of RF and XGBoost differed from that of the modified vergence formula (*P* = 0.016 and 0.045, respectively). All the other *P* values of the Friedman test were > 0.05. The percentages of PEs within ± 0.25 D, ± 0.50 D, and ± 0.75 D were similar (Cochran Q test in Additional file 2) All the *P* values of the Cochran Q test were > 0.05 in all groups. The result of the normality test before and after adjusting MPE to zero (Kolmogorov-Smirnov test) is also shown in Additional file 3. The PE distribution of our stacking ML models (RF, LASSO, and SVR) was normal in NT-ICL cases (*P* values of Kolmogorov-Smirnov test were > 0.05), and the PE distribution in other conditions was not normal. The distribution of PE of MVF and our stacking ML models in NT-ICL and TICL cases are shown in Additional files 4 and 5, respectively. The mean, SD, MAE, MAD, kurtosis, skewness (asymmetry), and Geary ratio are shown on each graph. The SDs and MAEs of ML models were lower than that of MVF, similar to the result after adjustment. The Bland-Altman plot showed strong agreement between the predictive values by our optimal stacking ML models and MVF (Fig. 3). The mean differences were all close to zero (all *P* values > 0.05). Over 95% of the data points were within the limits of agreement in all the groups.

**Table 2:** Prediction performance of the stacking machine learning models in the testing set

Parameters	SD (D)	MAE (D)	MedAE (D)	Interquartile of AE (D)	Percentage of eyes within the ranges (%)		
					± 0.25 D	± 0.5 D	± 0.75 D
Postoperative SE prediction of NT-ICL cases (n = 130)							
MVF	0.525	0.385	0.310	0.391	43.08	70.77	87.69
Random forest	**0.445**	**0.339**	**0.268**	**0.372**	48.46	73.08	89.23
LASSO	0.448	0.347	0.303	0.399	44.62	74.62	91.54
SVR	0.477	0.359	0.275	0.415	48.46	73.08	88.46
XGBoost	0.485	0.385	0.305	0.402	43.08	70.77	87.69
Postoperative sphere prediction of NT-ICL cases (n = 130)							
MVF	**0.485**	0.392	0.335	0.375	36.15	73.85	84.62
Random forest	0.489	**0.386**	0.336	**0.312**	40.77	70.00	86.15
LASSO	0.487	0.387	**0.312**	0.347	41.54	64.62	88.46
SVR	0.486	**0.386**	0.347	0.328	40.00	67.69	87.69
XGBoost	0.513	0.403	0.328	0.391	36.92	70.77	86.15
Postoperative SE prediction of TICL cases (n = 205)							
MVF	0.470	0.341	0.269	**0.311**	47.32	80.48	92.68
Random forest	0.462	0.333	0.264	0.320	48.78	80.49	90.24
LASSO	0.457	0.337	0.275	0.333	47.32	78.54	94.15
SVR	0.461	0.337	0.271	0.336	46.83	79.02	93.66
XGBoost	**0.452**	**0.325**	**0.257**	0.316	50.24	79.51	92.20
Postoperative sphere prediction of TICL cases (n = 205)							
MVF	0.460	0.323	0.243	**0.328**	53.66	81.46	90.73
Random forest	0.447	0.313	0.232	0.351	54.15	81.46	92.20
LASSO	0.462	0.325	**0.231**	0.354	51.71	78.05	91.71
SVR	0.467	0.330	0.238	0.343	52.68	78.05	91.22
XGBoost	**0.443**	**0.308**	0.241	0.344	52.68	80.98	92.20

*NT-ICL* = non-toric implantable collamer lens; *TICL* = toric implantable collamer lens; *MAE* = mean absolute error; *MedAE* = median absolute error; *AE* = absolute error; *SD* = standard deviation; *D* = diopters; *MVF* = modified vergence formula; *SVR* = support vector regression

The mean prediction error was adjusted to zero in each subgroup. The numerically smallest SD, MAE, MedAE, and AE quartile are marked in bold for each subgroup. The *P* value of the prediction error distribution of postoperative spherical equivalent after TICL implantation was 0.014. The distribution of the prediction error of random forest and XGBoost differed from that of the modified vergence formula *P* = 0.016 and 0.045, respectively. All the other *P* values of the Friedman test were > 0.05

### Subgroup analysis

We compared the performance of the existing MVF and our stacking ML models for NT-ICL cases and TICL cases among patients with different myopia ranges in Tables 3 and 4, respectively. Numerically, our stacking ML models achieved lower MAEs, SDs, and MedAEs than MVF in patients with low-to-moderate myopia (preoperative SE >  − 6.00 D). The *P* values of the Friedman test were > 0.05 in all the groups. This advantage was not obvious in high myopia and super high myopia.

**Table 3:** Prediction performance in different levels of myopia in NT-ICL cases (n = 130)

Parameters	Preop SE > − 6.00 D (n = 20)				Preop SE − 6.00 to − 10.00 D (n = 70)				Preop SE < − 10.00 D (n = 40)			
	MPE (D)	SD (D)	MAE (D)	MedAE (D)	MPE (D)	SD (D)	MAE (D)	MedAE (D)	MPE (D)	SD (D)	MAE (D)	MedAE (D)
Postoperative SE prediction												
MVF	0.152	0.598	0.492	0.325	− 0.016	**0.455**	0.365	0.295	− 0.048	0.473	**0.389**	0.253
Random forest	0.006	0.566	0.426	0.382	− 0.028	0.476	**0.363**	**0.280**	0.046	0.481	0.404	0.240
LASSO	0.006	0.555	0.406	**0.279**	− 0.050	0.477	0.374	0.286	0.084	0.467	0.401	**0.209**
SVR	0.015	**0.553**	**0.403**	0.331	− 0.053	0.478	0.376	0.309	0.086	**0.463**	0.392	0.249
XGBoost	− 0.026	0.569	0.422	0.306	− 0.026	0.508	0.391	0.283	0.058	0.502	0.413	0.217
Postoperative sphere prediction												
MVF	− 0.135	0.604	0.471	0.445	0.047	0.459	0.364	0.300	− 0.015	0.590	0.380	**0.330**
Random forest	− 0.024	0.581	0.445	0.368	− 0.023	0.471	0.355	0.284	0.053	0.446	0.328	0.375
LASSO	− 0.022	0.569	0.420	0.310	− 0.040	**0.457**	**0.353**	**0.271**	0.082	**0.342**	**0.274**	0.357
SVR	− 0.027	**0.562**	**0.416**	**0.296**	− 0.039	0.465	0.363	0.347	0.081	0.343	0.284	0.380
XGBoost	− 0.075	0.567	0.427	0.455	− 0.008	0.484	0.366	0.306	0.052	0.421	0.312	0.419

*NT-ICL* = non-toric implantable collamer lens; *MPE* = mean prediction error; *MAE* = mean absolute error; *MedAE* = median absolute error; *SD* = standard deviation; *D* = diopters; *MVF* = modified vergence formula; *SVR* = support vector regression. The numerically smallest SD, MAE and MedAE are marked in bold for each subgroup

**Table 4:** Prediction performance in different levels of myopia in TICL cases (n = 205)

Parameters	Preop SE > − 6.00 D (n = 18)				Preop SE − 6.00 to − 10.00 D (n = 93)				Preop SE < − 10.00 D (n = 94)			
	MPE (D)	SD (D)	MAE (D)	MedAE (D)	MPE (D)	SD (D)	MAE (D)	MedAE (D)	MPE (D)	SD (D)	MAE (D)	MedAE (D)
Postoperative SE prediction												
MVF	0.166	0.459	0.289	0.171	− 0.039	0.365	0.284	0.251	0.007	0.563	0.408	0.293
Random forest	− 0.080	0.376	0.245	0.146	0.016	0.351	0.274	0.247	− 0.001	0.565	0.407	0.322
LASSO	− 0.046	**0.281**	**0.199**	0.113	0.003	0.362	0.283	0.245	0.006	0.560	0.417	0.347
SVR	− 0.038	0.291	**0.199**	**0.089**	0.003	0.355	0.281	**0.240**	0.004	0.570	0.418	0.330
XGBoost	− 0.069	0.361	0.240	0.149	0.015	**0.348**	**0.271**	0.257	− 0.002	**0.552**	**0.395**	**0.290**
Postoperative sphere prediction												
MVF	− 0.178	0.254	0.236	0.183	0.021	0.367	0.283	0.247	0.013	0.559	0.379	**0.250**
Random forest	− 0.036	0.252	0.198	0.155	0.008	**0.349**	**0.269**	**0.208**	− 0.001	0.553	0.378	0.254
LASSO	− 0.033	**0.241**	**0.182**	0.144	− 0.007	0.378	0.289	0.235	0.014	0.561	0.389	0.275
SVR	− 0.008	0.256	0.194	0.162	− 0.007	0.380	0.290	0.225	0.009	0.569	0.397	0.295
XGBoost	− 0.056	0.242	0.188	**0.141**	0.011	0.354	0.272	0.236	− 0.001	**0.543**	**0.366**	0.264

*TICL* = toric implantable collamer lens; *MPE* = mean prediction error; *MAE* = mean absolute error; *MedAE* = median absolute error; *SD* = standard deviation; *D* = diopters; *MVF* = modified vergence formula; *SVR* = support vector regression. The numerically smallest SD, MAE and MedAE are marked in bold for each subgroup

### Application of lens power calculator

**Fig. 4:**
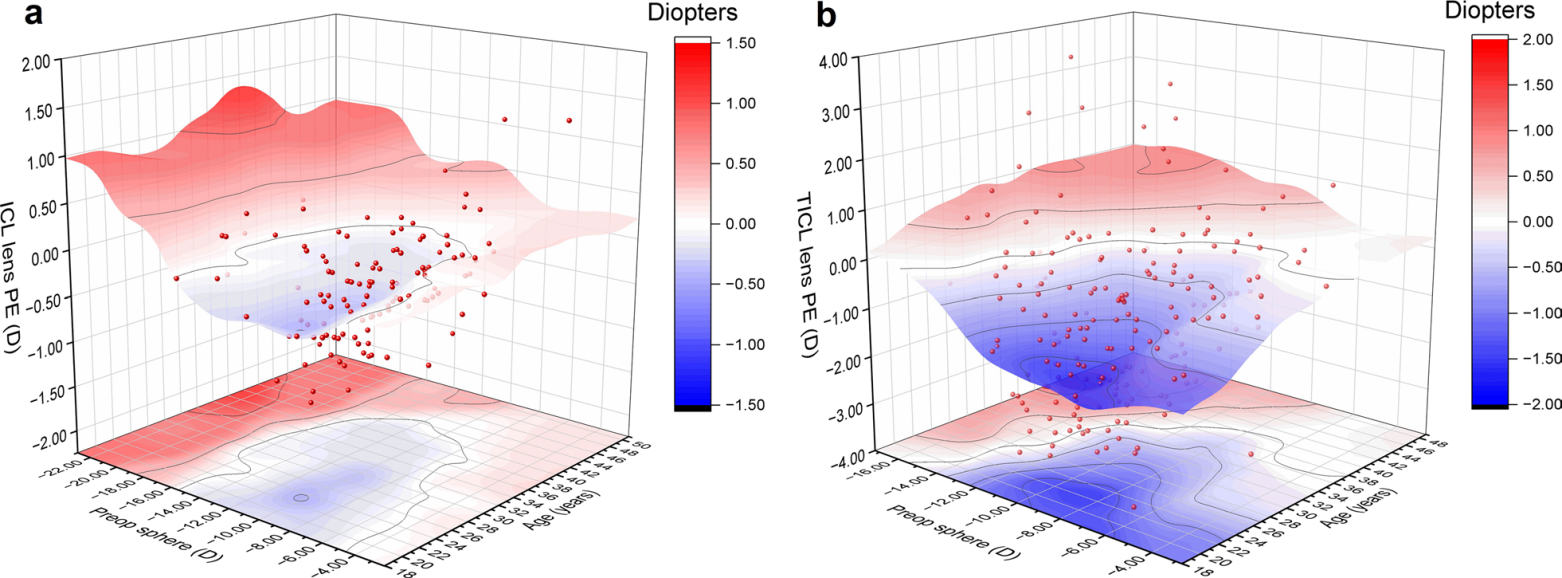
The 3D surface of non-toric implantable collamer lens (NT-ICL) and toric implantable collamer lens (TICL) sphere prediction errors. **a** NT-ICL sphere prediction errors. **b** TICL sphere prediction errors. A 3D surface representation of regions where the recommended lens sphere by stacking ML models differs from the implanted lens sphere. The lens prediction error was calculated by subtracting the recommended lens sphere (when the predicted postoperative sphere was closest to zero) from the implanted lens sphere. Blue represents a negative value (myopic shift), and red represents a positive value (hyperopic shift). 3D, three dimensional; PE, prediction error; preop, preoperative

Table 5 demonstrates the disparity between the recommended and implanted lens sphere values when targeting emmetropia. The disparity of NT-ICL did not differ significantly from 0, while that of TICL was lower with − 0.729 ± 1.420 D than implanted power (Table 5). The 3D surface (Fig. 4) highlights the areas of clinical disparity between the optimal stacking ML models and the MVF. The myopic shift tended to be larger for younger patients than for older patients, and a hyperopic shift tended to occur in individuals with more severe myopia (> − 16.00 D).

**Table 5:** Disparity between the recommended and implanted lens sphere values when targeting emmetropia

Parameters	MPE (D)	Range of PE (D)	SD (D)	MAE (D)	MedAE (D)	Percentage of eyes within the ranges (%)		
						± 0.25 D	± 0.5 D	± 0.75 D
NT-ICL lens sphere	0.016	− 1.25 to 1.50	0.981	0.347	0.25	59.50	85.95	94.21
TICL lens sphere	− 0.729^a^	3.25 to 2.50	1.420	1.221	1.00	23.98	33.16	45.41

*PE* = prediction error; *MPE* = mean prediction error; *MAE* = mean absolute error, *MedAE* = median absolute error; *SD* = standard deviation; *D* = diopters

The disparity between the recommended power and implanted lens power of NT-ICL did not differ significantly from zero, while that of TICL was lower with − 0.729 D than the implanted power. The recommended lens sphere deviated from the implanted lens sphere by < 0.25 D (one step provided by the manufacturer) in 56.92% of NT-ICL cases and < 0.50 D (one step provided by the manufacturer) in 32.20% of the time in TICL cases

^a^Statistically significant difference from zero (*P* < 0.05)

## Discussion

The customized lens power calculation is crucial for ensuring satisfactory visual outcomes, thereby achieving successful refractive surgery. Our optimal stacking ML models achieved numerically lower MAE and SD than the MVF and demonstrated strong agreement with MVF. The optimal ML model yielded 48.46% and 40.77% of eyes with a predictive error within 0.25 D, which was approximately 4% and 5% more than the OCOS calculator of STAAR company. Our results demonstrated that surgeons could use stacking ML models to combine various ocular parameters for each case, achieving comparable accuracy to MVF in predicting postoperative sphere and SE. Furthermore, in low-to-moderate myopia (preoperative SE >  − 6.00 D), the MAE difference between our ML models and MVF was more obvious, indicating that stacking ML models may have a potential advantage in low-to-moderate myopia. For clinical application, we developed an ICL power calculator displaying the predicted ICL powers for ICL lens power selection.

According to the Van der Heijde vergence formula, phakic IOL lens power is theoretically calculated based on preoperative manifest refraction, keratometry, and expected lens position [27]. The present study involved preoperative manifest refraction, age, and ocular dimensional parameters (keratometry, AL, IOP, ACV, ACA, ACD, scotopic PD, and Pentacam PD) as predictors of postoperative refractive errors. As expected, preoperative refraction errors (SE and sphere) were the most important factor in predicting postoperative refractive outcomes. The crystalline lens in phakic eyes after ICL implantation retains accommodation function to compensate for the postoperative refractive error caused by the change of ICL position. Thus, we considered that the recovery of accommodation function affects postoperative refractive errors in phakic eyes, especially in the early postoperative period [28]. Previous studies reported that postoperative intended refractive errors varied with the severity of myopia [17–19], and higher myopia (with an SE ≤  − 6.00 D) tends to have worse accommodation function[17]. Since there are far more patients with high myopia (preoperative SE − 6.00 to − 10.00 D), the MAEs of MVF and ML models were lower in the high myopia group in NT-ICL cases. Contrarily, the MAEs were smaller in the low-to-moderate myopia group(previous SE >  − 6.00 D) in TICL cases.

Age is another important variable affecting accommodation function [29] and showed a certain correlation with the postoperative outcomes in our study. Luo et al. found that vision and accommodative functions improved significantly after ICL implantation in younger patients than in patients aged over 35 years [30]. Here, we observed that older patients usually have less accommodation during manifest refraction than younger patients, which may cause more stable preoperative and postoperative refraction evaluations. Nevertheless, our findings showed that the preoperative manifest refraction should be accurately determined.

The AL seems to have a significant impact on predicting postoperative refraction. Additional file 6 shows that the MAE and SD are smaller in AL, between 26 and 30 mm, which the larger sample size can explain in the moderately long AL. Previous studies with existing IOL formulas demonstrated strong correlations between AL and PE. In our study, only the PE of postoperative SE for NT-ICL by MVF was statistically correlated with AL (r =  − 0.222, *P* = 0.011). The fact that ICL is implanted in the sulcus but not the capsular bag explains this. The difference of MAE between MVF and ML models was larger in extreme AL (less than 26 mm and over 30 mm), which shows that ML-based methods have the potential to better capture the nonlinearity of the relationship between biometric variables, ICL power, and the postoperative refractive refraction, resulting in substantially smaller AL bias.

Herein, various ocular dimensional parameters (IOP, ACV, ACA, scotopic PD, and Pentacam PD) were involved in our models because of their potential association with the postoperative lens position [31, 32]. Previous studies have observed a tendency toward a hyperopic shift with a higher vault and vice versa [14–16, 33]. With the vault higher, the distance of ICL and ocular posterior polar increases so that ICL lens power increases and vice versa. In our study, we analyzed the correlation between PE and vault (Additional file 7). The result showed that PE of the MVF was significantly hyperopic (PE > 0.00 D) with a higher vault and myopic (PE < 0.00 D) with a lower vault (postoperative ICL SE: r = 0.245, *P* = 0.007; postoperative ICL sphere: r = 0.237, *P* = 0.009; postoperative TICL SE: r = 0.153, *P* = 0.03; postoperative TICL sphere: r = 0.142, *P* = 0.004). Our ML models demonstrated a flatter slope than MVF. However, the correlation did not reach statistical significance (SVR for ICL SE: r = 0.128, *P* = 0.161; XGBoost for TICL SE: r = 0.124, *P* = 0.079; XGBoost for TICL sphere: r = 0.114, *P* = 0.107), except for that of postoperative SE prediction after ICL by stacking RF (r = 0.206, *P* = 0.023). The result showed that our ML models might correct the vault bias by incorporating the ocular dimensional parameters related to the vault. Considering that we must enter preoperative parameters to predict postoperative refraction error in clinical applications and the limited accuracy of vault prediction, we did not include vault into our ML model.

Interestingly, the importance of these ocular dimensional parameters weighed heavily in the TICL dataset, which means that the postoperative refractive errors after TICL implantation was affected by more factors, including the preoperative lens power calculation and postoperative rotation [34, 35]. We found that the MAEs in the low and moderate myopia subgroups were smaller than those in the high and super high myopia. The difference in ocular structure in low and high myopia can explain this. The higher levels of myopia have a weaker ocular structure, such as a larger sulcus diameter, which may lead to less vault and easier rotation. The correlation of vault change and rotation was also observed in previous studies [36, 37]. Park et al. found that the absolute value of rotation was correlated with the spherical power of TICL [38]. The spherical power of TICL may increase its thickness and height, and the cylinder power of TICL may increase its asymmetry, which may contribute to its postoperative rotation. In addition, a large pupil size (over 4 mm) may influence the prolate or aspheric shape of the cornea, which may cause the overcorrection of astigmatism [39]. Further studies with larger samples are needed to explore the relationship between ocular dimensional parameters and postoperative astigmatism in the TICL model.

Regarding the application of our model, we evaluated the most appropriate lens sphere for targeting emmetropia by enumerating the lens power 0.25 D step. The divergence between the NT-ICL and implanted lens power did not differ significantly from zero, showing that our ML models and the MVF are alternatives for the calculation of NT-ICL lens sphere. However, the recommended TICL sphere was lower by − 0.729 D than the implanted TICL sphere, which would cause a myopic shift, which may be explained by the larger interval of the TICL sphere provided by the manufacturer (0.50 D) than our interval (0.25 D). However, the result of divergence between recommended and implanted power reflects differences in clinical selection. However, it does not mean that our formula is not sufficiently accurate. Age and preoperative SE may affect the clinical selection. When designing the lens power, younger patients (under 30 years) tended to leave hyperopia. Contrarily, elder patients (over 40 years) tended to leave myopia for monovision design [40]. A hyperopic shift was more likely to occur in individuals with more severe myopia (> − 16.00 D), which may be because of the upper limit of the lens sphere (− 18.00 D) provided by the manufacturer. In clinical application, the predicted postoperative SE and corresponding ICL or NT-ICL spheres can be demonstrated for surgeons, making it convenient to adjust the lens power according to the needs and targets of each patient. Future studies are needed to explore a more personalized lens power design for different age groups and preoperative power groups.

The accuracy of our ML models can be attributed to the stacking ML technique, which is one of the strengths of our study. In addition, we trained ML models based on the merging data and the separate NT-ICL or TICL datasets to capture similar and different factors influencing postoperative refractive error. Thus, we provided a reliable result and a novel perspective on factors of postoperative refraction by exploring the interpretability of the ML model.

This study had some limitations. First, our study used data from a single center. Our data remains to be validated with an external multicenter dataset. Second, we did not predict the postoperative cylinder or calculate the TICL cylinder because more complex models are needed to perform vector analysis. Our ML models for TICL showed a relatively weaker correlation with MVF and a larger disparity of lens sphere. Third, we excluded the patients with a myopic target (patients with presbyopia or monovision surgeries) when analyzing the result of recommended lens power (Table 5). Since we did not involve the target refractive error when calculating our model’s lens power, the target refractive error varies with individuals. Recommended lens power has to be adjusted manually according to the predictive result in patients with monovision design. Finally, we used data obtained over a short period postoperatively. ICL implantation through a 3-mm corneal incision has a negligible effect on the refractive outcome and is less subject to the wound-healing response of the cornea. We included the cases of two experienced surgeons (XYW and XTZ) performed with over 1,000 eyes of ICL implantation to ensure the surgical quality and minimize the other causes of refractive instability. In our clinical experience, the perioperative medication stopped one week after surgery, and the effect of viscosurgical device disappeared after one day postoperatively. Our previous study found that patients with high myopia showed continuous myopic progression and axial elongation at an adult age one year after ICL surgery [41]. We did not use long-term visual outcomes. In the future, we will introduce external data, combine more parameters, and explore models for predicting astigmatism with wider applications and higher accuracy. The longer period of follow-up data will also be included to predict the refractive stability after ICL surgery.

## Conclusions

Ocular power and position during implantation may affect postoperative refraction error after ICL implantation. With various ocular dimensional parameters, ML models demonstrate comparable accuracy to the existing MVF and potential advantages in low-to-moderate myopia providing a novel nomogram for postoperative refractive error prediction and lens power calculation.

## Supplementary Information

**Additional file 1**: Friedman test results of the distribution of prediction error and absolute error.

**Additional file 2**. Cochran Q test results of the percentage of prediction errors within ranges.

**Additional file 3**. Normality test of prediction error in test dataset.

**Additional file 4**. The prediction error distribution before adjustment in the test dataset of NT-ICL cases.

**Additional file 5**. The prediction error distribution before adjustment in the test dataset of TICL cases.

**Additional file 6**. Prediction performance in different ranges of axial length.

**Additional file 7**. Correlation between vault and prediction error (PE).

## Data Availability

The datasets during and analyzed in the current study are available from the corresponding author upon reasonable request.

## Abbreviations

IOL: Intraocular lenses
ICL: Implantable collamer lens
NT-ICL: Non-toric implantable collamer lens
TICL: Toric implantable collamer lens
D: Diopters
AI: Artificial intelligence
ML: Machine learning
UDVA: Uncorrected distance visual acuity
BDVA: Best-corrected distance visual acuity
AL: Axial length
IOP: Intraocular pressure
K1: Steepest keratometry
K2: Flattest keratometry
ACD: Anterior chamber depth
ACA: Anterior chamber angle
PD: Pupil diameter
CT: Corneal thickness
WTW: White-to-white
SE: Spherical equivalent
SVR: Support vector regression
MVF: Modified vergence formula
PE: Prediction error
MPE: Mean prediction error
SD: Standard deviation
MAE: Mean absolute error
MedAE: Median absolute error
3D: Three-dimensional
