# Supplementary material for: Artificial intelligence-based refractive error prediction and EVO-implantable collamer lens power calculation for myopia correction
Source: Eye Vis (Lond). 2023 May 1;10:22. doi: 10.1186/s40662-023-00338-1 (PMC10150472; doi:10.1186/s40662-023-00338-1)
Supplement: Supplementary file 1 — Additional file 1: Friedman test results of the distribution of prediction error and absolute error. [file 40662_2023_338_MOESM1_ESM.docx]

**Additional file 1. Friedman test results of the distribution of prediction error and absolute error**

| **Parameters** | **Statistic** | ***P* value** |
| --- | --- | --- |
| PE of the postoperative SE after NT-ICL implantation | 4.695 | 0.32 |
| PE of the postoperative sphere after NT-ICL implantation | 0.695 | 0.952 |
| PE of the postoperative SE after TICL implantation | 12.57 | 0.014a |
| PE of the postoperative sphere after TICL implantation | 3.301 | 0.509 |
| AE of the postoperative SE after NT-ICL implantation | 8.34 | 0.08 |
| AE of the postoperative sphere after NT-ICL implantation | 6.222 | 0.183 |
| AE of the postoperative SE after TICL implantation | 2.868 | 0.58 |
| AE of the postoperative sphere after TICL implantation | 1.319 | 0.858 |

NT-ICL = non-toric implantable collamer lens; TICL = toric implantable collamer lens; PE = prediction error; AE = absolute error; SE = spherical equivalent.

According to the post hoc test, the distribution of the prediction error of random forest and XGBoost differed from that of the modified vergence formula (*P* = 0.016 and 0.045, respectively).
