# Supplementary material for: Artificial intelligence-based refractive error prediction and EVO-implantable collamer lens power calculation for myopia correction
Source: Eye Vis (Lond). 2023 May 1;10:22. doi: 10.1186/s40662-023-00338-1 (PMC10150472; doi:10.1186/s40662-023-00338-1)
Supplement: Supplementary file 2 — Additional file 2. Cochran Q test results of the percentage of prediction errors within ranges. [file 40662_2023_338_MOESM2_ESM.docx]

**Additional file 2. Cochran Q test results of the percentage of prediction errors within ranges**

| **Parameters** | **Statistic** | ***P* value** |
| --- | --- | --- |
| Postoperative SE prediction after NT-ICL implantation |  |  |
| %Rx within ±0.25 D | 3.304 | 0.508 |
| %Rx within ±0.5 D | 1.524 | 0.822 |
| %Rx within ±0.75 D | 3.182 | 0.528 |
| Postoperative sphere prediction after NT-ICL implantation |  |  |
| %Rx within ±0.25 D | 3.055 | 0.549 |
| %Rx within ±0.5 D | 7.769 | 0.100 |
| %Rx within ±0.75 D | 3.040 | 0.551 |
| Postoperative SE prediction after TICL implantation |  |  |
| %Rx within ±0.25 D | 1.652 | 0.799 |
| %Rx within ±0.5 D | 1.488 | 0.829 |
| %Rx within ±0.75 D | 8.818 | 0.066 |
| Postoperative sphere prediction after TICL implantation |  |  |
| %Rx within ±0.25 D | 0.822 | 0.936 |
| %Rx within ±0. 5 D | 6.136 | 0.189 |
| %Rx within ±0.75 D | 1.789 | 0.774 |

SE = spherical equivalent; NT-ICL = non-toric implantable collamer lens; TICL = toric implantable collamer lens; %Rx = percentage of the predicted error; D = diopters
