# Supplementary material for: Artificial intelligence-based refractive error prediction and EVO-implantable collamer lens power calculation for myopia correction
Source: Eye Vis (Lond). 2023 May 1;10:22. doi: 10.1186/s40662-023-00338-1 (PMC10150472; doi:10.1186/s40662-023-00338-1)
Supplement: Supplementary file 3 — Additional file 3. Normality test of prediction error in test dataset. [file 40662_2023_338_MOESM3_ESM.docx]

| **Additional File 3. Normality test of prediction error in test dataset** | | | | | | | | |
| --- | --- | --- | --- | --- | --- | --- | --- | --- |
|  | **Statistic** | ***P* value** | **Decision at level (5%)** |  | **Statistic** | ***P* value** | **Decision at level (5%)** |  |
| **Prediction error of postoperative SE in NT-ICL test dataset (n=130 eyes)** | | | | | | | | |
| MVF | 0.120 | 0.000 | Reject normality |  | 0.064 | 0.200 | Can't reject normality |  |
| Random forest | 0.071 | 0.188 | Can't reject normality |  | 0.043 | 0.200 | Can't reject normality |  |
| Lasso | 0.067 | 0.200 | Can't reject normality |  | 0.041 | 0.200 | Can't reject normality |  |
| SVR | 0.065 | 0.200 | Can't reject normality |  | 0.063 | 0.200 | Can't reject normality |  |
| XGBoost | 0.080 | 0.038 | Reject normality |  | 0.051 | 0.200 | Can't reject normality |  |
| **Prediction error of postoperative sphere in NT-ICL test dataset (n=130 eyes)** | | | | | | | | |
| MVF | 0.064 | 0.200 | Can't reject normality |  | 0.119 | 0.000 | Reject normality |  |
| Random forest | 0.041 | 0.200 | Can't reject normality |  | 0.067 | 0.200 | Can't reject normality |  |
| Lasso | 0.043 | 0.200 | Can't reject normality |  | 0.071 | 0.188 | Can't reject normality |  |
| SVR | 0.063 | 0.200 | Can't reject normality |  | 0.065 | 0.200 | Can't reject normality |  |
| XGBoost | 0.051 | 0.200 | Can't reject normality |  | 0.080 | 0.038 | Reject normality |  |
| **Prediction error of postoperative SE in TICL test dataset (n=205 eyes)** | | | | | | | | |
| MVF | 0.088 | 0.001 | Reject normality |  | 0.088 | 0.001 | Reject normality |  |
| Random forest | 0.107 | <0.001 | Reject normality |  | 0.071 | 0.013 | Reject normality |  |
| Lasso | 0.071 | 0.013 | Reject normality |  | 0.107 | <0.001 | Reject normality |  |
| SVR | 0.087 | 0.001 | Reject normality |  | 0.087 | 0.001 | Reject normality |  |
| XGBoost | 0.093 | <0.001 | Reject normality |  | 0.093 | <0.001 | Reject normality |  |
| **Prediction error of postoperative sphere in TICL test dataset (n=205 eyes)** | | | | | | | | |
| MVF | 0.106 | <0.001 | Reject normality |  | 0.087 | 0.001 | Reject normality |  |
| Random forest | 0.092 | <0.001 | Reject normality |  | 0.092 | <0.001 | Reject normality |  |
| Lasso | 0.087 | 0.001 | Reject normality |  | 0.090 | <0.001 | Reject normality |  |
| SVR | 0.090 | <0.001 | Reject normality |  | 0.113 | <0.001 | Reject normality |  |
| XGBoost | 0.113 | <0.001 | Reject normality |  | 0.106 | <0.001 | Reject normality |  |

NT-ICL = non-toric implantable collamer lens; TICL = toric implantable collamer lens; MVF = modified vergence formula; SVR = support vector regression

The normality of the prediction error was assessed using the Kolmogorov-Smirnov test. In the testing set of NT-ICL and TICL cases, we adjusted the ME of different models to 0 to eliminate the systematic error caused by the clinical environment.
