# Supplementary material for: Artificial intelligence-based refractive error prediction and EVO-implantable collamer lens power calculation for myopia correction
Source: Eye Vis (Lond). 2023 May 1;10:22. doi: 10.1186/s40662-023-00338-1 (PMC10150472; doi:10.1186/s40662-023-00338-1)
Supplement: Supplementary file 4 — Additional file 4. The prediction error distribution before adjustment in the test dataset of NT-ICL cases. [file 40662_2023_338_MOESM4_ESM.docx]

**
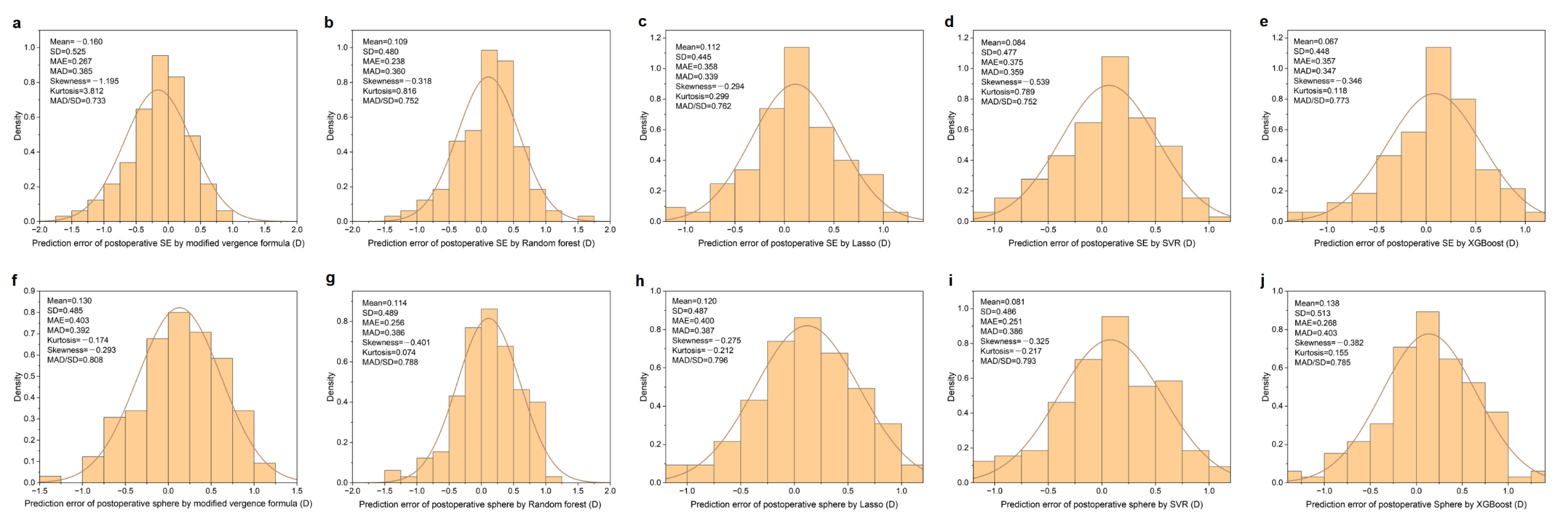
Additional file 4. The prediction error distribution before adjustment in the test dataset of NT-ICL cases.** The mean, SD, MAE, MAD, kurtosis, skewness (asymmetry), and Geary ratio are shown in each graph. The number of y-axis is probability density function that x falls into a certain interval. The SDs and MAEs of the ML models were lower than those of MVF, which was similar to the result after adjustment. The skewness and kurtosis of postoperative SE prediction error by the ML models (random forest: skewness = −0.318, kurtosis = −0.816) were closer to zero than MVF (skewness = −1.195, kurtosis = −3.812), which means more symmetric distribution of PE and the lower extremity of deviations in predicting postoperative refraction error by the ML models in NT-ICL cases. NT-ICL, non-toric implantable collamer lens; SE, spherical equivalent; SD, standard deviation; ML, machine learning; MAE, mean absolute error; MAD, mean absolute deviation; MVF, modified vergence formula; SVR, support vector regression; D, diopters.
