# Supplementary material for: Artificial intelligence-based refractive error prediction and EVO-implantable collamer lens power calculation for myopia correction
Source: Eye Vis (Lond). 2023 May 1;10:22. doi: 10.1186/s40662-023-00338-1 (PMC10150472; doi:10.1186/s40662-023-00338-1)
Supplement: Supplementary file 5 — Additional file 5. The prediction error distribution before adjustment in the test dataset of TICL cases. [file 40662_2023_338_MOESM5_ESM.docx]

#
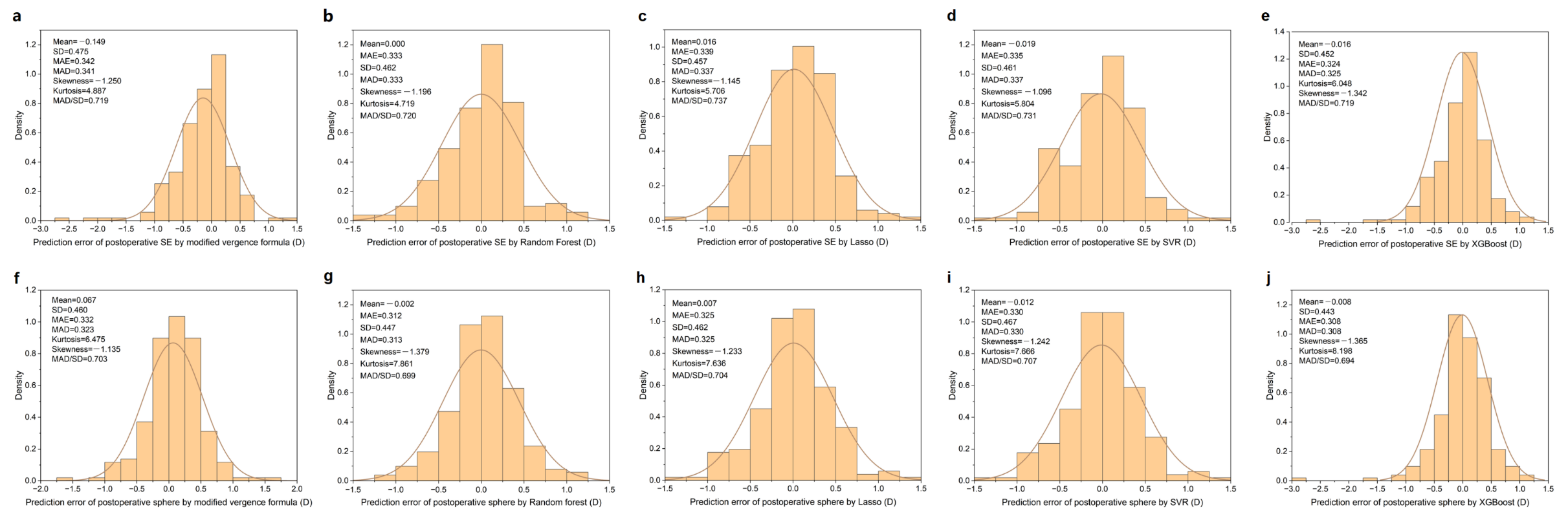
Additional file 5. The prediction error distribution before adjustment in the test dataset of TICL cases. The mean, SD, MAE, MAD, kurtosis, skewness (asymmetry), and Geary ratio are shown in each graph. The number of y-axis is probability density function that x falls into a certain interval. The SDs and MAEs of the ML models were lower than those of MVF, which was similar to the result after adjustment. The skewness and kurtosis were similar in the ML models and MVF. TICL, toric implantable collamer lens; SE, spherical equivalent; SD, standard deviation; ML, machine learning; MAE, mean absolute error; MAD, mean absolute deviation; MVF, modified vergence formula; SVR, support vector regression; D, diopters.
