# Supplementary material for: Artificial intelligence-based refractive error prediction and EVO-implantable collamer lens power calculation for myopia correction
Source: Eye Vis (Lond). 2023 May 1;10:22. doi: 10.1186/s40662-023-00338-1 (PMC10150472; doi:10.1186/s40662-023-00338-1)
Supplement: Supplementary file 6 — Additional file 6. Prediction performance in different ranges of axial length. [file 40662_2023_338_MOESM6_ESM.docx]

**Additional file 6. Prediction performance in different ranges of axial length**

|  | **AL < 26 mm (n=32)** | | | | | **AL 26****–28 mm (n=64)** | | | | | **AL 28–30 mm (n=28)** | | | | | | | | | | | | | **AL > 30 mm (n=6)** | | | | | | | | | | | |  |
| --- | --- | --- | --- | --- | --- | --- | --- | --- | --- | --- | --- | --- | --- | --- | --- | --- | --- | --- | --- | --- | --- | --- | --- | --- | --- | --- | --- | --- | --- | --- | --- | --- | --- | --- | --- | --- |
|  | **MPE** | **SD** | | **MAE** | **MedAE** | **MPE** | **SD** | **MAE** | | **MedAE** | | **MPE** | | | | **SD** | | | **MAE** | | **MedAE** | | | | **MPE** | | **SD** | | | **MAE** | | | **MedAE** | | | |
| **Postoperative SE prediction of NT-ICL cases (n=130)** | | | | | | | | | | | | | | | | | | | | | | | | | | | | | | | | | | | | |
| MVF | −0.082 | 0.585 | | 0.462 | 0.325 | 0.111 | 0.432 | 0.352 | | 0.305 | 0.032 | | | | 0.334 | | 0.264 | | | 0.245 | | | | −0.899 | | 0.926 | | | 0.899 | | | 0.745 | | | |  |
| Random forest | −0.004 | 0.556 | | 0.433 | 0.369 | 0.038 | 0.461 | 0.345 | | 0.316 | 0.007 | | | | 0.387 | | 0.268 | | | 0.209 | | | | −0.417 | | 0.548 | | | 0.571 | | | 0.560 | | | |  |
| LASSO | −0.031 | 0.531 | | 0.392 | 0.284 | 0.023 | 0.454 | 0.358 | | 0.292 | 0.002 | | | | 0.365 | | 0.284 | | | 0.193 | | | | −0.089 | | 0.132 | | | 0.114 | | | 0.101 | | | |  |
| SVR | −0.031 | 0.528 | | 0.398 | 0.315 | 0.011 | 0.452 | 0.355 | | 0.342 | 0.038 | | | | 0.379 | | 0.293 | | | 0.208 | | | | −0.125 | | 0.273 | | | 0.234 | | | 0.212 | | | |  |
| XGBoost | −0.036 | 0.543 | | 0.418 | 0.312 | 0.060 | 0.444 | 0.346 | | 0.261 | −0.002 | | | | 0.407 | | 0.279 | | | 0.190 | | | | −0.439 | | 0.618 | | | 0.558 | | | 0.471 | | | |  |
| **Postoperative SE prediction of NT-ICL cases (n=130)** | | | | | | | | | | | | | | | | | | | | | | | | | | | | | | | | | | | | |
| MVF | 0.096 | 0.599 | | 0.488 | 0.440 | −0.087 | 0.440 | 0.365 | | 0.325 | −0.038 | | | | 0.363 | | 0.298 | | | 0.290 | | | | 0.597 | | 0.311 | | | 0.597 | | | 0.710 | | | |  |
| Random forest | 0.022 | 0.569 | | 0.452 | 0.402 | 0.019 | 0.453 | 0.359 | | 0.307 | 0.011 | | | | 0.460 | | 0.346 | | | 0.287 | | | | −0.376 | | 0.503 | | | 0.503 | | | 0.383 | | | |  |
| LASSO | 0.002 | 0.550 | | 0.427 | 0.341 | 0.009 | 0.470 | 0.375 | | 0.271 | 0.016 | | | | 0.460 | | 0.360 | | | 0.258 | | | | −0.189 | | 0.512 | | | 0.443 | | | 0.381 | | | |  |
| SVR | 0.006 | 0.547 | | 0.424 | 0.380 | −0.011 | 0.475 | 0.385 | | 0.347 | 0.062 | | | | 0.442 | | 0.343 | | | 0.296 | | | | −0.199 | | 0.511 | | | 0.387 | | | 0.281 | | | |  |
| XGBoost | 0.004 | 0.591 | | 0.464 | 0.441 | 0.036 | 0.491 | 0.383 | | 0.306 | −0.017 | | | | 0.458 | | 0.358 | | | 0.263 | | | | −0.331 | | 0.563 | | | 0.497 | | | 0.402 | | | |  |
|  | **AL < 26 mm (n=32)** | | | | | **AL 26–28 mm (n=113)** | | | | | **AL 28–30 mm (n=53)** | | | | | | | | | | | | | **AL > 30 mm (n=7)** | | | | | | | | | | | |  |
|  | **MPE** | | **SD** | **MAE** | **MedAE** | **MPE** | **SD** | | **MAE** | **MedAE** | | | **MPE** | **SD** | | | | **MAE** | | | | **MedAE** | **MPE** | | | | | **SD** | | | **MAE** | | | **MedAE** |  |  |
| **Postoperative SE prediction of TICL cases (n=205)** | | | | | | | | | | | | | | | | | | | | | | | | | | | | | | | | | | | | |
| MVF | 0.110 | 0.510 | | 0.346 | 0.221 | −0.053 | 0.388 | 0.300 | | 0.246 | 0.023 | | | | 0.583 | | 0.402 | | | 0.299 | | | | 0.181 | | 0.634 | | | 0.522 | | | 0.641 | | | |  |
| Random forest | −0.116 | 0.428 | | 0.311 | 0.232 | 0.034 | 0.395 | 0.300 | | 0.222 | −0.018 | | | | 0.591 | | 0.405 | | | 0.318 | | | | 0.119 | | 0.516 | | | 0.422 | | | 0.412 | | | |  |
| LASSO | −0.111 | 0.388 | | 0.289 | 0.216 | 0.033 | 0.396 | 0.311 | | 0.261 | −0.052 | | | | 0.576 | | 0.393 | | | 0.263 | | | | 0.362 | | 0.527 | | | 0.550 | | | 0.521 | | | |  |
| SVR | −0.099 | 0.393 | | 0.286 | 0.243 | 0.031 | 0.392 | 0.308 | | 0.256 | −0.060 | | | | 0.576 | | 0.391 | | | 0.275 | | | | 0.415 | | 0.610 | | | 0.618 | | | 0.567 | | | |  |
| XGBoost | −0.120 | 0.414 | | 0.293 | 0.230 | 0.048 | 0.381 | 0.296 | | 0.231 | −0.033 | | | | 0.587 | | 0.397 | | | 0.290 | | | | 0.028 | | 0.488 | | | 0.391 | | | 0.324 | | | |  |
| **Postoperative sphere prediction of TICL cases (n=205)** | | | | | | | | | | | | | | | | | | | | | | | | | | | | | | | | | | | | |
| MVF | −0.127 | 0.429 | | 0.340 | 0.252 | 0.055 | 0.377 | 0.279 | | 0.223 | −0.012 | | | | 0.597 | | 0.388 | | | 0.253 | | | | −0.222 | | 0.531 | | | 0.464 | | | 0.563 | | | |  |
| Random forest | −0.108 | 0.374 | | 0.301 | 0.245 | 0.038 | 0.367 | 0.266 | | 0.183 | −0.022 | | | | 0.609 | | 0.401 | | | 0.280 | | | | 0.055 | | 0.497 | | | 0.439 | | | 0.458 | | | |  |
| LASSO | −0.113 | 0.385 | | 0.311 | 0.260 | 0.032 | 0.390 | 0.283 | | 0.193 | −0.043 | | | | 0.598 | | 0.394 | | | 0.254 | | | | 0.324 | | 0.561 | | | 0.549 | | | 0.470 | | | |  |
| SVR | −0.084 | 0.395 | | 0.313 | 0.238 | 0.031 | 0.398 | 0.291 | | 0.177 | −0.059 | | | | 0.598 | | 0.395 | | | 0.254 | | | | 0.324 | | 0.579 | | | 0.553 | | | 0.426 | | | |  |
| XGBoost | −0.121 | 0.373 | | 0.299 | 0.236 | 0.050 | 0.370 | 0.269 | | 0.211 | −0.034 | | | | 0.594 | | 0.385 | | | 0.261 | | | | 0.004 | | 0.450 | | | 0.391 | | | 0.349 | | | |  |

AL = axial length; NT-ICL = non-toric implantable collamer lens; TICL = toric implantable collamer lens; MPE = mean prediction error; MAE = mean absolute prediction error; MedAE = median absolute prediction error; SD = standard deviation; SE = spherical equivalent; MVF = modified vergence formula; SVR = support vector regression

The MAE and SD are smaller in AL, between 26 and 30 mm, which the larger sample size can explain in the moderately long AL. The prediction error of postoperative SE for NT-ICL by MVF was statistically correlated with AL (R = −0.222, *P* = 0.011).
