# Supplementary material for: Artificial intelligence-based refractive error prediction and EVO-implantable collamer lens power calculation for myopia correction
Source: Eye Vis (Lond). 2023 May 1;10:22. doi: 10.1186/s40662-023-00338-1 (PMC10150472; doi:10.1186/s40662-023-00338-1)
Supplement: Supplementary file 7 — Additional file 7. Correlation between vault and prediction error (PE). [file 40662_2023_338_MOESM7_ESM.docx]

#
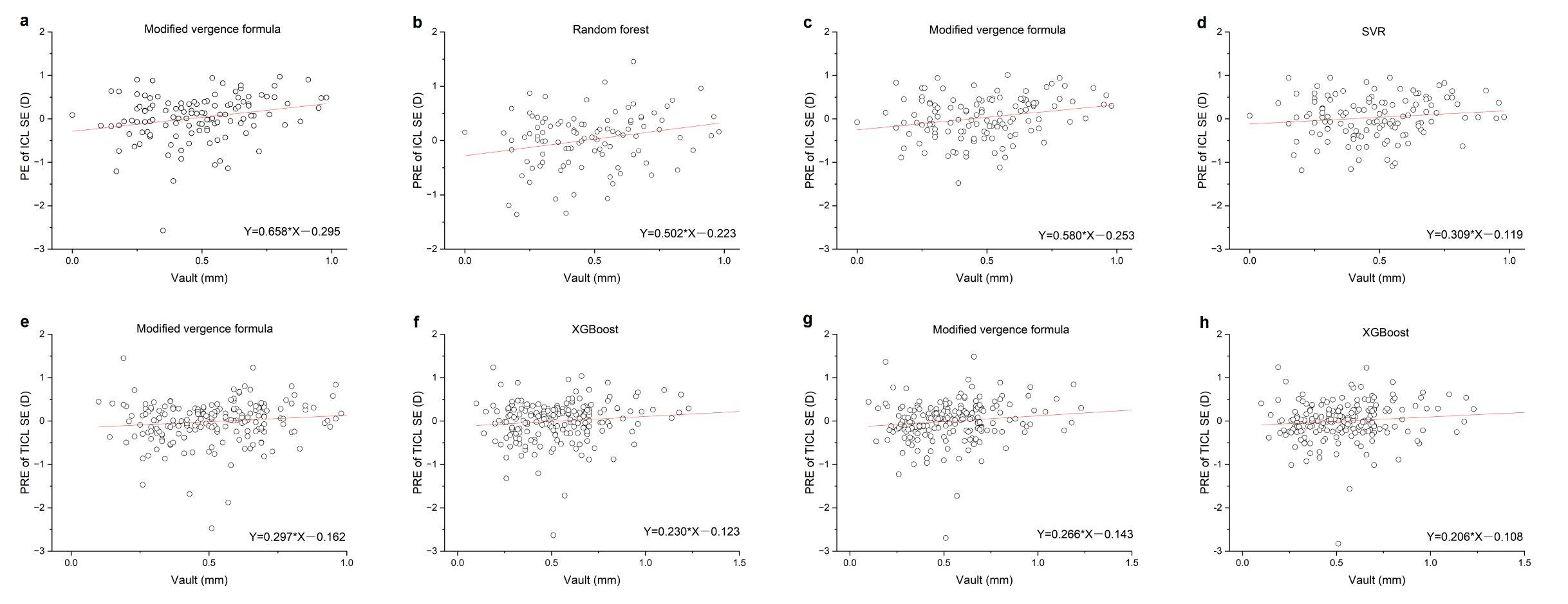
Additional file 7. Correlation between vault and prediction error (PE). PE of the modified vergence formula (MVF) was significantly hyperopic (PE > 0) with a higher vault and myopia (PE < 0) with a lower vault (postoperative implantable collamer lens (ICL) spherical equivalent (SE): r = 0.245, *P* = 0.007; postoperative ICL sphere: r = 0.237, *P* = 0.009; postoperative toric ICL (TICL) SE: r = 0.153, *P*=0.03; postoperative TICL sphere: r = 0.142, *P* = 0.004). Our machine learning models demonstrated a flatter slope than MVF. The correlation did not reach statistical significance (support vector regression [SVR] for ICL SE: r = 0.128, *P* = 0.161; XGBoost for TICL SE: r = 0.124, *P* = 0.079; XGBoost for TICL sphere: r = 0.114, *P* = 0.107), except for that of postoperative SE prediction after ICL by stacking random forest (r = 0.206, *P* = 0.023).
